# Spatial analysis of granting of social welfare benefits to people with gastrointestinal Chagas disease in Brazil, 2004-2016: a time series study

**DOI:** 10.1590/S2237-96222025v34e20240622.en

**Published:** 2025-06-13

**Authors:** Jean Ezequiel Limongi, Keile Aparecida Resende Santos, Izabela Lima Perissato, Rivaldo Mauro Faria

**Affiliations:** 1 Universidade Federal de Uberlândia, Instituto de Geografia, Geociências e Saúde Coletiva. Uberlândia, MG, Brazil; 2 Instituto Nacional do Seguro Social. Uberlândia, MG, Brazil

**Keywords:** Chagas Disease, Social Support, Social Security, Geographic Mapping, Time Factors, Enfermedad de Chagas, Apoyo Social, Seguridad Social, Mapeo Geográfico, Factores de Tiempo

## Abstract

**Objective:**

To analyze patterns of spatial association in the granting of social welfare benefits to individuals with gastrointestinal Chagas disease in Brazil in the period 2004-2016.

**Methods:**

This was a time series analysis, using secondary data provided by the Ministry of Labor and Employment. The analyses were performed using global and local Moran indices spatial autocorrelation techniques.

**Results:**

In all, 4,661 benefits were granted, mainly to residents of urban areas (n=3,285, 70.5%), males (n=2,819, 60.5%) and with average age of 49.5±9.3 years. The main benefits granted were social welfare due to temporary incapacity (n=3,754, 80.5%), retirement due to permanent incapacity (n=581, 12.5%) and assistance support for people with disabilities (n=320, 6.9%). The most significant values ​​found for the global Moran index were for the variables “benefits to individuals aged ≥60 years old” (0.673; p-value 0.001) and “benefits granted in urban areas” (0.666; p-value 0.001). Health macro-regions in the states of Minas Gerais, Goiás and Bahia stood out, forming high-high clusters in the local Moran index analysis when benefit granting was analyzed according to sex, area of ​​residence, type of benefit granted and age.

**Conclusion:**

The concentration of benefit granting in Minas Gerais, Goiás and Bahia is a result of the intense transmission of the disease in these areas in past decades. Longitudinal monitoring of the population chronically affected by Chagas disease, mainly by Primary Care teams, can reduce the impact of the disease on social support and social security.

## Resumen

**Objetivo:**

Analizar patrones de asociación espacial en la concesión de prestaciones de seguridad social a personas con enfermedad de Chagas digestiva en Brasil en el período 2004-2016.

**Métodos:**

Se realizó un análisis de series de tiempo, utilizando datos secundarios del Ministerio de Trabajo y Empleo. Los análisis se llevaron a cabo utilizando técnicas de autocorrelación espacial de índices de Moran global y local.

**Resultados:**

Se otorgaron 4.661 beneficios, principalmente a residentes de áreas urbanas (n=3.285, 70,5%), hombres (n=2.819, 60,5%) y con edad promedio de 49,5±9,3 años. Las principales prestaciones concedidas fueron asistencia social por incapacidad temporal (n=3.754, 80,5%), jubilación por incapacidad permanente (n=581, 12,5%) y apoyo asistencial a personas con discapacidad (n=320, 6,9%). Los valores más significativos encontrados para el índice de Moran global fueron para las variables “beneficios a personas ≥60 años” (0,673; p-valor 0,001) y “beneficios otorgados en áreas urbanas” (0,666; p-valor 0,001). Se destacaron las macrorregiones de salud de los estados de Minas Gerais, Goiás y Bahía, formando conglomerados (clusters) del tipo alto-alto en el análisis del índice de Moran local cuando se analizó la concesión de prestaciones según sexo, zona de residencia, tipo de prestación concedida y edad.

**Conclusión:**

La concentración de la concesión de beneficios en Minas Gerais, Goiás y Bahía es resultado de la intensa transmisión de la enfermedad en esas áreas en décadas pasadas. El seguimiento longitudinal de la población afectada crónicamente por la enfermedad de Chagas, principalmente por parte de los equipos de Atención Primaria, puede reducir el impacto de la enfermedad en la atención sanitaria y la seguridad social.

**Ethical aspects**

This research respected ethical principles, having obtained the following approval data:

Research Ethics Committee: Universidade Federal de Uberlândia

Opinion number: 1,560,139/2016

Approval date: 17/5/2016

Certificate of Submission for Ethical Appraisal: 52527516.4.0000.5152

Informed Consent Form: Not applicable.

## Introduction

Chagas disease is a neglected tropical disease, with chronic progression and high morbidity and mortality. The disease is endemic in 21 countries in America and it is estimated that, worldwide, 7 million people may be infected with *Trypanosoma cruzi* (1,2). As cases become chronic, the predominant clinical forms of the disease emerge: cardiac, digestive, cardiodigestive and undetermined. Chronic Chagas heart disease is the most frequent, disabling and lethal disease. Digestive forms, represented mainly by megacolon and megaesophagus, cause symptoms that affect people’s quality of life. Examples of these symptoms are dysphagia, regurgitation, fear of eating, malnutrition, severe constipation and the presence of fecaloma (3).

Gastrointestinal involvement in Chagas disease is considered a neglected manifestation of a neglected disease (3-10). The prevalence of gastrointestinal manifestations in individuals with Chagas disease has been estimated at 12.0% (10). Diagnosis of this clinical form of Chagas disease is late, and treatment is generally inadequate and difficult to access, resulting in emergency surgeries (4). Between 2017 and 2019, more than 60.0% of hospitalizations for gastrointestinal manifestations of Chagas disease were emergencies; 50.0% of them resulted in surgical procedures. Hospital mortality was 5.8%, and in intensive care units it reached 17.2% (4). Longitudinal monitoring of Chagas disease patients in Primary Health Care has been recommended since 2015, but case management at this level of health care is incipient (3,11-14).

The worsening of these cases also impacts social security and social support, due to the need to grant social welfare benefits to workers and people in vulnerable conditions (15). Spatial analysis of the granting of these benefits helps in identifying priority territories for intervention.

This study aimed to analyze the patterns of spatial association in the granting of social welfare benefits to individuals with gastrointestinal Chagas disease in Brazil, between 2004 and 2016, with stratification of sociodemographic data.

## Methods

### Design

This is an exploratory study, of the time series analysis type, based on secondary data. We analyzed the distribution of social welfare benefits for people with gastrointestinal Chagas disease in Brazil between 2004 and 2016. 

### Setting

We analyzed sociodemographic data and data related to the granting of social security and social support benefits to individuals with gastrointestinal Chagas disease. All beneficiaries who received benefits granted by the National Social Security Institute between 2004 and 2016 were selected when they were registered with International Classification of Diseases Codes B57.3 (Chagas disease (chronic) with digestive system involvement), K23.1 (megaesophagus in Chagas disease) or K93.1 (megacolon in Chagas disease).

### Participants

People with gastrointestinal Chagas disease who were receiving social security or social support benefits from January 1, 2004 to December 31, 2016 participated in the study.

### Variables

The following variables were analyzed: sex (male, female), age (in years), age group (in years: ≤29, ≥60), area of ​​residence (urban, rural), type of benefit granted (social security, social support), age at disease onset (in years) and at onset of incapacity (in years), time elapsed between disease onset and incapacity onset (in months), regions of Brazil (North, Northeast, Midwest, South and Southeast) and health macro-regions. We took 119 Brazilian health macro-regions into account (16). The “sex” variable used biological distinction as its basis (17). The two age groups analyzed (in years: ≤29, ≥60) were used to verify places with the highest frequency of cases among the younger and older population.

### Data sources

The data source we used was the Ministry of Social Security Unified Benefits Information System, access to which is restricted. The system was developed by the Social Security Technology and Information Company. The system holds sociodemographic data on beneficiaries and data related to benefits granted. 

### Study size

The study covered data on all individuals who received social security or social support benefits in Brazil between 2004 and 2016, whose cause of incapacity for work was gastrointestinal Chagas disease. 

### Statistical methods

The analyses were performed using exploratory spatial data techniques, namely spatial autocorrelation, using global and local Moran indices (Moran’s I) (18). Global Moran’s I enables an overall measure of spatial association for the data set and can vary from -1 to +1, indicating spatial independence (when the value of the variable is zero and the null hypothesis of no spatial dependence is confirmed), direct spatial dependence (when the value is positive) or inverse spatial dependence (when the value is negative) (19). 

The global Moran’s I autocorrelation function provides a single spatial association value for the data set, which can conceal local (or regional) realities that are included in this single global value. We also decided to apply the Local Indicator of Spatial Association (LISA), or local Moran’s I, (18), in order to identify territorial areas where the indicator presents pronounced spatial dependence and tends to form clusters. The use of LISA enabled identification of spatial patterns that could later be compared. The global and local Moran’s I analyses were accompanied by the pseudo-significance test, without which it cannot be stated whether the data are random (stationary) or dependent (non-stationary). In this study, the null hypothesis of data randomness was rejected with a 99.0% confidence level in the aforementioned test. 

Data were analyzed according to the Moran scatterplot. Four quadrants are presented in the scatterplot: Q1 (high-high) and Q2 (low-low) represent positive spatial autocorrelation between the values ​​of a variable and the average of its neighbors, that is, the values ​​of a given indicator in one place are similar to the values ​​of neighboring places, forming so-called clusters. Quadrants Q3 (high-low) and Q4 (low-high) indicate negative spatial autocorrelation, that is, the values ​​of a variable in a given place are not spatially related to its neighbors. These cases are commonly assessed as situations of spatial stationarity, as they indicate places for which the value of a given indicator (high or low) is opposite to that presented by immediately nearby places. These atypical spatial situations are represented as outliers (19).

We used QGis, version 3.36.2, and GeoDa 1.22 to perform statistical operations and spatial representation of data. In the choropleth maps, the data were distributed into five classes, using the natural breaks method (20). The cartographic base, by municipalities, was obtained from the Brazilian Institute of Geography and Statistics, on the scale 1:25,000,000. From this database, and considering the list of municipalities belonging to each health macro-region, it was possible to model a new cartographic base with the health macro-region delimitations.

### Access to data cleansing and cleansing methods

Access to data occurred after a formal request made to the central management level of the National Social Security Institute. The data was made available in Excel® spreadsheet format, in January 2017. We performed an analysis of data completeness and consistency, before performing statistical analysis. The data were assessed descriptively, using percentages (for categorical variables), and measures of central tendency and dispersion (for numerical variables). Blank fields or fields with discrepant values ​​were checked: those with the possibility of correction, identified by analyzing the other variables, were changed; while the remainder were discarded from the specific analysis.

## Results

Between 2004 and 2016, 4,661 benefits were granted to Brazilian social welfare beneficiaries whose cause of incapacity for work was gastrointestinal Chagas disease. The benefits were granted mainly to residents of urban areas (n=3,285, 70.5%), males (n=2,819, 60.5%) and with an average age of 49.5±9.3 years (minimum 13 years, maximum 83 years). The main benefits granted were social welfare due to temporary incapacity (n=3,754, 80.5%), retirement due to permanent incapacity (n=581, 12.5%) and assistance support for people with disabilities (n=320, 6.9%). 

In the case of 2,888 (62.0%) benefits, only chronic Chagas disease was recorded, with digestive system involvement, without detailing the site. A further 1,219 (26.1%) were recorded as megaesophagus, and 554 (11.9%) as megacolon. The granting of benefits in Brazilian geographic regions had the following distribution: Southeast (n=1,976, 42.4%), Northeast (n=1,485, 31.9%), Midwest (n=896, 19.2%), South (n=235, 5.0%) and North (n=69, 1.5%).

The results found using global Moran’s I showed that the variables are spatially dependent. The most significant values ​​of positive spatial autocorrelation were found for the variables “benefits granted to individuals aged ≥60 years old” (I=0.673; p-value 0.001) and “benefits granted in urban areas” (I=0.666; p-value 0.001) (Table 1).

**Table 1:** Global Moran’s index values per variable analyzed. Brazil, 2004-2016 (n=4,661)

Variables analyzed	Global Moran’s index	p-value
Benefits granted (overall)	0.489	<0.001
Social support benefits granted	0.386	<0.001
Social security benefits granted	0.477	<0.001
Benefits granted to individuals aged ≤29 years old	0.220	<0.001
Benefits granted to individuals aged ≥60 years old	0.673	<0.001
Benefits granted in urban areas	0.666	<0.001
Benefits granted in rural areas	0.371	<0.001
Benefits granted to males	0.477	<0.001
Benefits granted to females	0.463	<0.001
Average age of beneficiaries at disease onset	0.275	<0.001
Average age of beneficiaries at onset of incapacity	0.176	<0.001
Average time until beneficiary incapacity for work	0.164	<0.001

**Figure 1:**
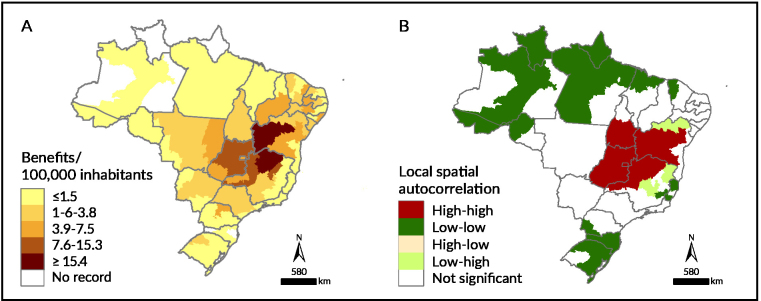
Rate of benefit granting to individuals affected by gastrointestinal Chagas disease (A) and local Moran index spatial autocorrelation (B). Brazil, 2004-2016 (n=4,661)

Local autocorrelation indicators were calculated for all study variables (Table 1). The highest concentrations of granted benefits were identified in health macro-regions in the states of Minas Gerais, Goiás and Bahia, with emphasis on the northern health macro-regions in Minas Gerais and north-central (Jacobina regional health district) and west (Barreiras primary health district) in Bahia (Figure 1A).

In the spatial autocorrelation analysis, clusters with a low-low pattern were observed distributed across four Brazilian regions, with the exception of the Midwest region. An extensive cluster of high-high patterns was formed in the central region of the country, encompassing 14 health macro-regions: southern macro-region in Tocantins; southwest (Vitória da Conquista primary health district), west (Barreiras primary health district), central-east (Feira de Santana regional health district) and central-north (Jacobina regional health district) macro-regions in Bahia; north, northwest and northern triangle macro-regions in Minas Gerais; and all macro-regions of Goiás and the Federal District (Figure 1B).

**Figure 2:**
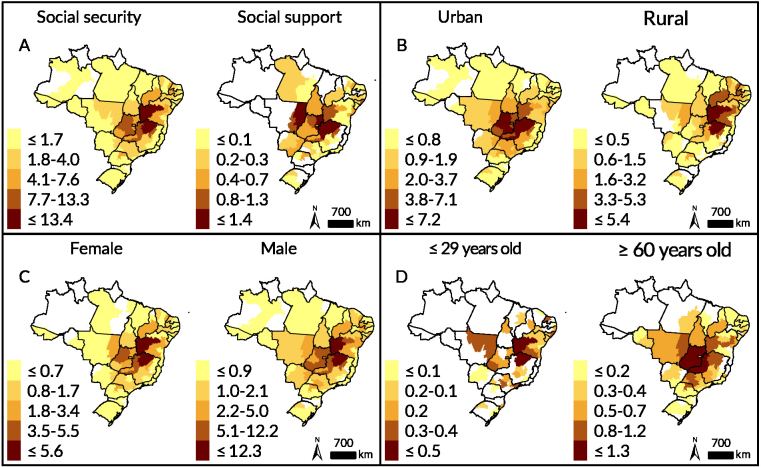
Rate of benefit granting (per 100,000 inhabitants) to individuals affected by gastrointestinal Chagas disease according to type of benefit (A), area of ​​residence (B), sex (C) and age (D). Brazil, 2004-2016 (n=4,661)

**Figure 3:**
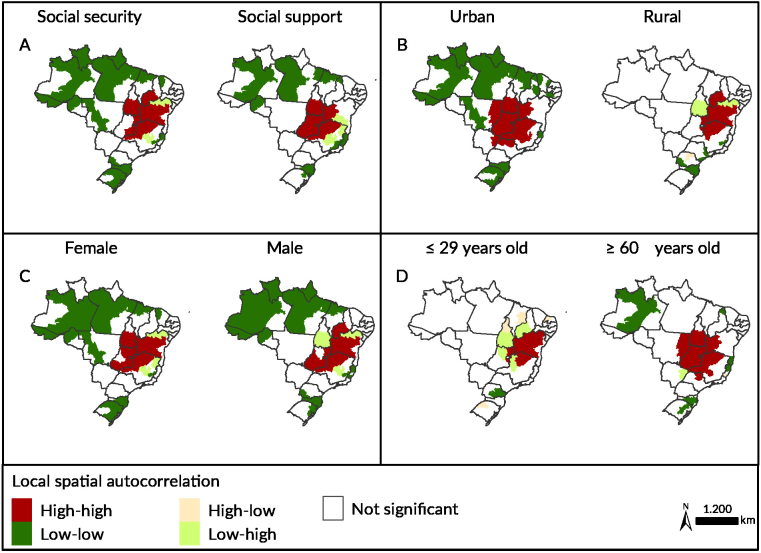
Local Moran index spatial autocorrelation for benefit granting to individuals affected by gastrointestinal Chagas disease according to type of benefit (A), area of ​​residence (B), sex (C) and age (D). Brazil, 2004-2016 (n=4,661)

The distribution of social security benefits was more frequent in health macro-regions of Minas Gerais, Goiás and Bahia. In the spatial autocorrelation, a high-high cluster pattern was observed advancing from the central region of Brazil to the North and Northeast regions, covering the north, northwest and northern triangle macroregions in Minas Gerais, the northeast and center-southeast macroregions in Goiás and the Federal District, the southern health macroregions in Tocantins, the cerrados region in Piauí, and the southwestern macroregions (Vitória da Conquista primary health district), west (Barreiras primary health district), center-east (Feira de Santana regional health district) and center-north (Jacobina regional health district) in Bahia (Figures 2A and 3A). Social support benefits had a more concentrated distribution in the central region of the country, and the high-high cluster pattern was formed only in this region, in the southern health macro-regions in Tocantins, west (Barreiras primary health district) in Bahia, in the north, northwest and northern triangle macro-regions in Minas Gerais, in addition to the whole of Goiás and the Federal District (Figures 2A and 3A).

Benefit distribution in urban areas was more concentrated in the central region of the country. An extensive high-high cluster was formed, encompassing 18 health macro-regions distributed across seven Brazilian states (Tocantins, Bahia, Minas Gerais, São Paulo, Mato Grosso, Mato Grosso do Sul and Goiás), in addition to the Federal District (Figures 2B and 2F). In relation to benefits granted to residents of rural areas, another cluster of the high-high type was observed, but located further to the northeast of the country. The southwest (Vitória da Conquista primary health district), west (Barreiras primary health district), central-east (Feira de Santana regional health district) and central-north (Jacobina regional health district) macro-regions in Bahia, the north and northwest macro-regions in Minas Gerais, the northeast macro-region in Goiás and the cerrados region in Piauí were part of this cluster (Figures 2B and 3B).

Benefit granting showed a similar pattern when considering the sex of the beneficiaries. In spatial autocorrelation, a very similar high-high pattern cluster was noted for both sexes, covering 13 health macro-regions in Minas Gerais, Goiás, Bahia and Tocantins, in addition to the Federal District when considering the female sex. Another 13 health macro-regions of Minas Gerais, Goiás, Bahia, Piauí and the Federal District were included, when considering the male sex, and 11 of them were the same in both maps (Figures 2C and 3C).

The rate of benefits granted to individuals aged ≤29 years old was the lowest among all the variables analyzed. The highest concentrations of benefits for individuals in this age group were observed in Minas Gerais and Bahia (Figure 2D). A high-high type cluster was formed encompassing the northern macro-region in Minas Gerais, the northeast in Goiás and the southwest (Vitória da Conquista primary health district), west (Barreiras primary health district), center-east (Feira de Santana regional health district), center-north (Jacobina regional health district) and north (Juazeiro regional health district) macro-regions in Bahia (Figure 3D). Distribution of benefits to individuals aged ≥60 years old was more concentrated in the center of the country, with higher frequencies in Goiás and Minas Gerais (Figure 2D). An extensive cluster was formed between 15 health macro-regions in Tocantins, Bahia, Minas Gerais, São Paulo, Mato Grosso, Goiás and the Federal District (Figure 3D).

**Figure 4:**
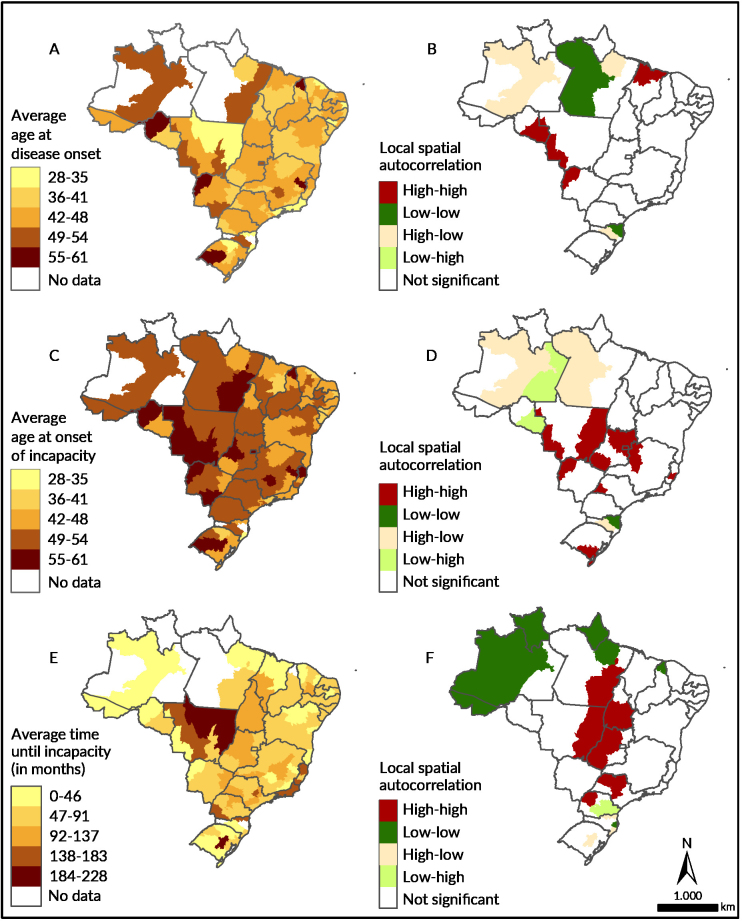
Average age at disease onset (A), at onset of incapacity (C) and average time until incapacity (E) for Brazilian social welfare beneficiaries affected by gastrointestinal Chagas disease and local Moran index spatial autocorrelation for average age at disease onset (B), at onset of incapacity (D) and average time until incapacity (F). Brazil, 2004-2016 (n=4,661)

Average age at disease onset, as well as average age at onset of incapacity for work and the average time until incapacity for work, showed a more dispersed cluster formation pattern, this being expected due to the low values ​​presented in the global Moran’s I (Figures 4A to 4F, Table 1). The low-low pattern clusters for the average time until incapacity for work were observed mainly in the Northern region of the country. The high-high pattern was observed in 11 macro-regions of six different states – São Paulo, Mato Grosso, Tocantins, Goiás, Pará and Paraná (Figure 3F).

## Discussion

The analysis showed that, in the period 2004-2016, Brazilian social welfare beneficiaries with gastrointestinal Chagas disease were mainly male, residing in urban areas, in the Southeast and Northeast regions of the country and aged between 50 and 59 years. Health macro-regions in Minas Gerais, Goiás and Bahia stood out, forming high-high clusters in the local Moran’s I analysis when benefit granting was analyzed according to sex, area of ​​residence, type of benefit granted and age. The Federal District also stood out in the composition of these clusters, being absent only in the high-high clusters related to residents of rural areas and those aged ≤29 years old. Low-low clusters formed mainly in the Northern and Southern regions of the country.

Using the General Social Security Regime database imposed an important limitation on the study. The case series analyzed only included individuals with Chagas disease enrolled in this Regime’s social security system or those who met the basic requirements to be entitled to social support benefits. This condition led to an underestimation of the number of workers affected by the disease, as individuals working in the informal sector were not included, nor were those subject to other social security schemes. Another important limitation was related to data timeliness, since this study only has information on Chagas disease in social welfare until 2016. Although the data used in this study were collected almost a decade ago, they are national in scope and cover the 12-year period of records on gastrointestinal Chagas disease and social welfare, serving as a basis for comparative analyses on the topic in future studies.

Most studies related to Chagas disease focus mainly on the cardiac form of the disease. There is a great epidemiological silence related to the gastrointestinal Chagas disease and its real prevalence, people’s quality of life, its potential for resulting in incapacity for work, among other issues. Despite the lower mortality of the digestive form compared to the cardiac form, morbidity caused by gastrointestinal manifestations of Chagas disease is considerable (10). There was an increase in mortality from digestive Chagas disease between 2000 and 2010. This fact could be related to the addition of a specific code for Chagas disease with digestive system involvement in the tenth edition of the International Classification of Diseases (21).

A total of 4,661 social welfare benefits related to digestive Chagas disease over the 12-year period in Brazil is a relatively small number. The arguments provided below may be able to explain this.

1) Digestive Chagas disease accounts for 12% prevalence among total Chagas disease cases (10). This prevalence rate may be underestimated, since both in cases of megaesophagus and megacolon forms of the disease, diagnosis is often not performed (8,9). People seek care for megacolon later than for megaesophagus due to greater tolerance to intestinal constipation in relation to dysphagia. Many people only seek treatment when complications such as fecaloma and volvulus arise (22). Although megacolon may be more common than megaesophagus, it may not lead to as many hospitalizations or may simply be more difficult to diagnose. The most common complaint of megaesophagus is dysphagia, which may occur earlier in the course of the disease or be more noticeable than megacolon constipation, which, as a result, ends up being less investigated (4). The study of the digestive form of Chagas disease in rural populations, for example, has always been difficult due to the lack of X-ray equipment (8).
2) Chagas disease incidence, in general, has decreased drastically in recent decades, not affecting so strongly people of working age as it did in the 1960s, 1970s, 1980s and 1990s.
3) Gastrointestinal Chagas disease is a neglected manifestation of a neglected disease, lacking more careful attention from academia and public health, which can conceal many chronic cases (4).

Due to inadequate and late diagnosis and treatment, individuals often develop more serious forms of the disease, resulting in incapacity for work. Hospitalizations commonly result in emergency surgeries (4). Although people with gastrointestinal Chagas disease have a longer survival rate when compared to those affected by the cardiac form, more advanced cases of “megas” present a high degree of morbidity and considerable loss of quality of life (23). Gastrointestinal Chagas disease generally affects individuals belonging to more vulnerable populations, who use public health services and, as a result, face major challenges in accessing healthcare (24). 

Spatial analysis showed that people with gastrointestinal Chagas disease are still concentrated in the classic areas of Chagas disease transmission – Goiás, Minas Gerais and Bahia. There has been intense migration of infected individuals from rural areas to cities. It can be seen that dispersion was not great among these individuals, as they are still concentrated in the same territories, but now in urban areas (11,25,26).

The majority of benefits were granted to individuals residing in urban areas. In Brazil, it was estimated in 2014 that 70% to 90% of people with T. cruzi infection lived in urban areas, mainly elderly people who were born in rural areas, where they were probably infected (27).

The average age of beneficiaries was 49.5±9.3 years. This data is consistent with the clinical picture of the disease, since, in the chronic form of gastrointestinal Chagas disease, individuals develop symptoms between the third and fifth decades of life, worsening over time (4). Successful vector control in the 1980s and 1990s in Brazil drastically reduced the incidence of the disease, reflected in this study by the low number of young beneficiaries (11). Bahia concentrated the largest number of beneficiaries under 30 years of age. This fact was also observed among individuals hospitalized because of chagasic megaesophagus, where 82.1% of cases of young individuals (under 31 years of age) came from Bahia (28). This fact may be related to the sanitary measures to control triatominae in Brazil, implemented in 1975, but without full coverage of the endemic area. Some areas of Bahia, for example, were not covered by the program during the initial stages, and risk of transmission there remained high. Only after 1983 was the entire Chagas disease endemic area covered by the national vector control program (28,29). 

The concentration of benefit granting in Minas Gerais, Goiás and Bahia is a result of the intense transmission of Chagas disease in these areas in past decades. Incapacity for work among the population chronically affected by Chagas disease can be reduced if there is longitudinal monitoring of individuals, preferably by Primary Health Care teams, reducing the impact of the disease on social support and social security.

## Data Availability

The database and the analysis codes used in this research are available at https://doi.org/10.48331/scielodata.KEK6F(30).
