# Supplementary material for: Spatial analysis of granting of social welfare benefits to people with gastrointestinal Chagas disease in Brazil, 2004-2016: a time series study
Source: Epidemiol Serv Saude. 2025 Jun 13;34:e20240622. doi: 10.1590/S2237-96222025v34e20240622.en (PMC12176446; doi:10.1590/S2237-96222025v34e20240622.en)
Supplement: Translation

# Translation: Análise espacial da concessão de benefícios da seguridade social para portadores da doença de Chagas digestiva no Brasil, 2004-2016: série temporal

**DOI:** 10.1590/S2237-96222025v34e20240622.pt

**Authors:** Jean Ezequiel Limongi, Keile Aparecida Resende Santos, Izabela Lima Perissato, Rivaldo Mauro Faria

**Affiliations:** 1 Universidade Federal de Uberlândia, Instituto de Geografia, Geociências e Saúde Coletiva. Uberlândia, MG, Brasil; 2 Instituto Nacional do Seguro Social. Uberlândia, MG, Brasil

**Keywords:** Doença de Chagas, Apoio Social, Previdência Social, Mapeamento Geográfico, Fatores de Tempo

## Abstract

**Objetivo:**

Analisar padrões de associação espacial da concessão de benefícios da seguridade social para indivíduos com doença de Chagas digestiva no Brasil no período 2004-2016.

**Métodos:**

Tratou-se de análise de série temporal, utilizando dados secundários do Ministério do Trabalho e Emprego. As análises foram realizadas com o uso das técnicas de autocorrelação espacial índices de Moran global e local.

**Resultados:**

Foram concedidos 4.661 benefícios, principalmente para moradores da zona urbana (n=3.285, 70,5%), do sexo masculino (n=2.819, 60,5%) e com idade média 49,5±9,3 anos. Os principais benefícios concedidos foram o auxílio por incapacidade temporária (n=3.754, 80,5%), a aposentadoria por incapacidade permanente (n=581, 12,5%) e o amparo assistencial ao portador de deficiência (n=320, 6,9%). Os valores mais expressivos encontrados no índice de Moran global foram para as variáveis “benefícios para indivíduos com idade ≥60 anos” (0,673; p-valor 0,001) e “benefícios concedidos em área urbana” (0,666; p-valor 0,001). Macrorregiões de saúde de Minas Gerais, de Goiás e da Bahia se destacaram formando aglomerados do tipo alto-alto na análise do índice de Moran local quando a concessão de benefícios foi analisada segundo sexo, área de residência, tipo de benefício concedido e idade.

**Conclusão:**

A concentração da concessão de benefícios em Minas Gerais, em Goiás e na Bahia é resultado da intensa transmissão da doença nessas áreas em décadas passadas. O acompanhamento longitudinal da população cronicamente afetada pela doença de Chagas, principalmente por equipes da Atenção Primária, pode diminuir o impacto da doença na assistência e na previdência social.

**Aspectos éticos**

A presente pesquisa respeitou os princípios éticos, obtendo os seguintes dados de aprovação: :

Comitê de ética em pesquisa: Universidade Federal de Uberlândia

Número do parecer: 1.560.139/2016

Data de aprovação: 17/5/2016

Certificado de apresentação de apreciação ética: 52527516.4.0000.5152

Registro do consentimento livre e esclarecido: Não se aplica.

## Introdução

A doença de Chagas é uma doença tropical negligenciada, de evolução crônica e elevada morbimortalidade. A doença é endêmica em 21 países da América e estima-se que, no mundo, 7 milhões de pessoas podem estar infectadas pelo *Trypanosoma cruzi* (1,2). Com a cronificação dos casos, surgem as formas clínicas predominantes da doença: a cardíaca, a digestiva, a cardiodigestiva e a indeterminada. A cardiopatia chagásica crônica é a mais frequente, incapacitante e letal da doença. As formas digestivas, representadas principalmente pelo megacólon e pelo megaesôfago, causam sintomas que afetam a qualidade de vida das pessoas. São exemplos desses sintomas a disfagia, a regurgitação, o medo de comer, a desnutrição, a prisão de ventre severa e a presença de fecaloma (3).

O envolvimento gastrointestinal na doença de Chagas é considerado uma manifestação negligenciada de uma doença negligenciada (3-10). A prevalência de manifestações gastrointestinais em indivíduos com doença de Chagas foi estimada em 12,0% (10). O diagnóstico dessa forma clínica da doença de Chagas é tardio, e o tratamento em geral é inadequado e de difícil acesso, resultando em cirurgias de emergência (4). Entre 2017 e 2019, mais de 60,0% das hospitalizações por manifestações gastrointestinais da doença de Chagas foram de emergência; 50,0% delas resultaram em procedimento cirúrgico. A mortalidade hospitalar foi 5,8%, e, em unidades de terapia intensiva, chegou a 17,2% (4). O acompanhamento longitudinal dos portadores de doença de Chagas na Atenção Primária à Saúde tem sido recomendado desde 2015, porém o manejo nesse nível de atenção à saúde ocorre de modo incipiente (3,11-14).

O agravamento desses casos impacta também a previdência e a assistência social, devido à necessidade de concessão de benefícios de seguridade social para trabalhadores e pessoas em condições de vulnerabilidade (15). A análise espacial relacionada à concessão desses benefícios auxilia na identificação de territórios prioritários para intervenção.

Este estudo teve como objetivo analisar os padrões de associação espacial da concessão de benefícios da seguridade social para indivíduos portadores da doença de Chagas digestiva no Brasil, entre 2004 e 2016, com estratificação de dados sociodemográficos.

## Métodos

### Delineamento

Trata-se de estudo exploratório, do tipo análise de série temporal, com base em dados secundários. Analisou-se a distribuição da concessão de benefícios da seguridade social para portadores da doença de Chagas digestiva no Brasil no período 2004-2016. 

### Contexto

Foram analisados dados sociodemográficos e relacionados às concessões de benefícios previdenciários e assistenciais para indivíduos portadores da doença de Chagas digestiva. Selecionaram-se todos os beneficiários que receberam benefícios concedidos pelo Instituto Nacional do Seguro Social entre 2004 e 2016, com o registro B57.3 (doença de Chagas crônica, com comprometimento do aparelho digestivo), K23.1 (megaesôfago na doença de Chagas) ou K93.1 (megacólon na doença de Chagas) na variável Código Internacional de Doenças.

### Participantes

Participaram do estudo portadores da doença de Chagas digestiva que estavam em gozo de benefícios previdenciários ou assistenciais de 1^o^ de janeiro de 2004 a 31 de dezembro de 2016.

### Variáveis

Analisaram-se as seguintes variáveis: sexo (masculino, feminino), idade (em anos), faixa etária (em anos: ≤29, ≥60), zona de residência (urbano, rural), tipo de benefício concedido (previdenciário, assistencial), idade no início da doença (em anos) e da incapacidade (em anos), tempo decorrido entre a doença e a incapacidade (em meses), regiões do Brasil (Norte, Nordeste, Centro-Oeste, Sul e Sudeste) e macrorregiões de saúde. Foram consideradas 119 macrorregiões de saúde brasileiras (16). A variável “sexo” manteve como base a distinção biológica (17). As duas faixas etárias analisadas (em anos: ≤29, ≥60) foram utilizadas para verificação dos locais com maior frequência de casos entre a população mais jovem e idosa. 

### Fontes de dados

A fonte de dados foi o Sistema Único de Informações de Benefícios do Ministério da Previdência Social, de acesso restrito, desenvolvido pela Empresa de Tecnologia e Informações da Previdência Social. Esse sistema possui dados sociodemográficos dos beneficiários e relacionados às concessões de benefícios. 

### Tamanho do estudo

O estudo abrangeu dados de todos os indivíduos que gozaram de benefícios da previdência ou assistência social no Brasil entre 2004 e 2016, cuja causa da incapacidade laboral foi a doença de Chagas digestiva. 

### Métodos estatísticos

As análises foram realizadas com uso das técnicas exploratórias de dados espaciais, nomeadamente a autocorrelação espacial, por meio dos índices de Moran (I de Moran) global e local (18). O I de Moran global permite uma medida geral de associação espacial para o conjunto dos dados e pode variar de -1 a +1, indicando independência espacial (quando o valor da variável é zero e a hipótese nula de não dependência espacial é confirmada), dependência espacial direta (quando o valor é positivo) ou inversa (quando o valor é negativo) (19). 

A função de autocorrelação do I de Moran global fornece único valor de associação espacial para o conjunto de dados, o que pode ocultar realidades locais (ou regionais) que estão subsumidas nesse único valor global. Decidiu-se também aplicar o indicador local de associação espacial (Local Indicators of Spatial Association – LISA), ou I de Moran local, (18), com intuito de identificar áreas territoriais onde o indicador apresenta dependência espacial pronunciada e tende à formação de aglomerados. O uso do LISA permitiu a identificação de padrões espaciais que, posteriormente, puderam ser comparados. As análises do I de Moran global e local foram acompanhadas do teste de pseudosignificância, sem o qual não se pode afirmar se os dados são randômicos (estacionários) ou dependentes (não estacionários). Neste estudo, a hipótese nula da aleatoriedade dos dados foi rejeitada com grau de confiança de 99,0% no referido teste. 

Os dados foram analisados de acordo com o diagrama de espalhamento de Moran. Nesse diagrama, quatro quadrantes são apresentados: Q1 (alto-alto) e Q2 (baixo-baixo) representam autocorrelação espacial positiva entre os valores de uma variável e a média dos vizinhos, ou seja, os valores de um dado indicador em uma localidade são similares aos valores de localidades vizinhas, formando os chamados aglomerados. Os quadrantes Q3 (alto-baixo) e Q4 (baixo-alto) indicam autocorrelação espacial negativa, ou seja, os valores de uma variável em determinada localidade não se relacionam espacialmente com os vizinhos. Esses casos são comumente avaliados como situações de estacionaridade espacial, pois indicam localidades cujo valor de um dado indicador (alto ou baixo) é oposto ao apresentado pelas localidades imediatamente próximas. Essas situações espaciais atípicas são representadas como discrepantes (19).

Para a realização das operações estatísticas e representação espacial dos dados, foram utilizados os programas QGis, versão 3.36.2, e o GeoDa 1.22. Nos mapas coropléticos, os dados foram distribuídos em cinco classes, por meio do método de quebras naturais (20). A base cartográfica, por municípios, foi retirada do Instituto Brasileiro de Geografia e Estatística, na escala utilizada 1:25.000.000. A partir dessa base, e considerando a relação dos municípios pertencentes a cada macrorregião de saúde, foi possível modelar uma nova base cartográfica com os limites das macrorregiões de saúde.

### Acesso aos dados e métodos de limpeza

O acesso aos dados ocorreu após solicitação formal ao nível central de gerência do Instituto Nacional de Seguro Social. Os dados foram disponibilizados em planilha Excel, em janeiro de 2017. Foi realizada a análise de completude e consistência dos dados, antes da análise estatística. Os dados foram avaliados de forma descritiva, por meio de porcentagens (para as variáveis categóricas), e medidas de tendência central e de dispersão (para as variáveis numéricas). Os campos em branco ou com valores discrepantes foram verificados: aqueles com possibilidade de correção, pela análise das demais variáveis, foram alterados; e os demais, descartados da análise específica.

## Resultados

Entre 2004 e 2016, 4.661 benefícios foram concedidos para beneficiários da seguridade social brasileira, cuja causa da incapacidade laboral foi a doença de Chagas digestiva. Os benefícios foram concedidos principalmente para moradores da zona urbana (n=3.285, 70,5%), do sexo masculino (n=2.819, 60,5%) e com idade média 49,5±9,3 anos (mínimo 13 anos, máximo 83 anos). Os principais benefícios concedidos foram o auxílio por incapacidade temporária (n=3.754, 80,5%), a aposentadoria por incapacidade permanente (n=581, 12,5%) e o amparo assistencial ao portador de deficiência (n=320, 6,9%). 

Em 2.888 (62,0%) benefícios, foi registrada apenas doença de Chagas crônica, com comprometimento do aparelho digestivo, sem detalhamento da localização. Outros 1.219 (26,1%) foram registrados como megaesôfago, e 554 (11,9%), como megacólon. A concessão de benefícios nas regiões geográficas brasileiras teve a seguinte distribuição: Sudeste (n=1.976, 42,4%), Nordeste (n=1.485, 31,9%), Centro-Oeste (n=896, 19,2%), Sul (n=235, 5,0%) e Norte (n=69, 1,5%).

Os resultados encontrados por meio do I de Moran global mostraram que as variáveis são espacialmente dependentes. Os valores mais expressivos de autocorrelação espacial positiva foram encontrados para as variáveis “benefícios concedidos para indivíduos com idade ≥60 anos” (I=0,673; p-valor 0,001) e “benefícios concedidos em área urbana” (I=0,666; p-valor 0,001) (Tabela 1).

**Tabela 1:** Valores do índice de Moran global por variável analisada. Brasil, 2004-2016 (n=4.661)

Variáveis analisadas	Índice de Moran global	p-valor
Benefícios concedidos (geral)	0,489	<0,001
Benefícios assistenciais concedidos	0,386	<0,001
Benefícios previdenciários concedidos	0,477	<0,001
Benefícios concedidos para indivíduos com idade ≤29 anos	0,220	<0,001
Benefícios concedidos para indivíduos com idade ≥60 anos	0,673	<0,001
Benefícios concedidos em área urbana	0,666	<0,001
Benefícios concedidos em área rural	0,371	<0,001
Benefícios concedidos para indivíduos do sexo masculino	0,477	<0,001
Benefícios concedidos para indivíduos do sexo feminino	0,463	<0,001
Média de idade dos beneficiários no início da doença	0,275	<0,001
Média de idade dos beneficiários no início da incapacidade	0,176	<0,001
Tempo médio até a incapacidade laboral dos beneficiários	0,164	<0,001

Foram calculados os indicadores de autocorrelação local para todas as variáveis do estudo (Tabela 1). Identificaram-se as maiores concentrações de concessões de benefícios em macrorregiões de saúde em Minas Gerais, em Goiás e na Bahia, com destaque para as macrorregiões de saúde norte em Minas Gerais e centro-norte (núcleo regional de saúde – Jacobina) e oeste (núcleo básico de saúde – Barreiras) na Bahia (Figura 1A). 

**Figura 1:** Taxa de concessão de benefícios para indivíduos acometidos pela doença de Chagas digestiva (A) e autocorrelação espacial do índice de Moran local (B). Brasil, 2004-2016 (n=4.661)

Na análise de autocorrelação espacial, os aglomerados de padrão baixo-baixo foram observados distribuídos em quatro regiões brasileiras, com exceção do Centro-Oeste. Um extenso aglomerado de padrão alto-alto se formou na região central do país, englobando 14 macrorregiões de saúde: macrorregião sul em Tocantins; macrorregiões sudoeste (núcleo básico de saúde – Vitória da Conquista), oeste (núcleo básico de saúde – Barreiras), centro-leste (núcleo regional de saúde – Feira de Santana) e centro-norte (núcleo regional de saúde – Jacobina) na Bahia; macrorregiões norte, noroeste e triângulo do norte em Minas Gerais; e todas as macrorregiões de Goiás e do Distrito Federal (Figura 1B). 

A distribuição de benefícios previdenciários foi mais frequente em macrorregiões de saúde de Minas Gerais, de Goiás e da Bahia. Na autocorrelação espacial, foi observado o padrão de aglomerado alto-alto avançando da região central do Brasil para as regiões Norte e Nordeste abrangendo as macrorregiões norte, noroeste e triângulo do norte em Minas Gerais, as macrorregiões nordeste e centro-sudeste em Goiás e no Distrito Federal, as macrorregiões de saúde sul em Tocantins, cerrados no Piauí, e as macrorregiões sudoeste (núcleo básico de saúde – Vitória da Conquista), oeste (núcleo básico de saúde – Barreiras), centro-leste (núcleo regional de saúde – Feira de Santana) e centro-norte (núcleo regional de saúde – Jacobina) na Bahia (Figuras 2A e 3A). Os benefícios assistenciais tiveram distribuição mais concentrada na região central do país, e o padrão de aglomerado alto-alto foi formado apenas nessa região, nas macrorregiões de saúde sul em Tocantins, oeste (núcleo básico de saúde – Barreiras) na Bahia, nas macrorregiões norte, noroeste e triângulo do norte em Minas Gerais, além de todo Goiás e o Distrito Federal (Figuras 2A e 3A).

A distribuição de benefícios em área urbana foi mais concentrada na região central do país. Um extenso aglomerado do tipo alto-alto foi formado, englobando 18 macrorregiões de saúde distribuídas em sete estados brasileiros (Tocantins, Bahia, Minas Gerais, São Paulo, Mato Grosso, Mato Grosso do Sul e Goiás), além do Distrito Federal (Figuras 2B e 2F). Em relação aos benefícios concedidos para moradores de áreas rurais, foi observado um outro aglomerado do tipo alto-alto, porém com localização mais ao nordeste do país. As macrorregiões sudoeste (núcleo básico de saúde – Vitória da Conquista), oeste (núcleo básico de saúde – Barreiras), centro-leste (núcleo regional de saúde – Feira de Santana) e centro-norte (núcleo regional de saúde – Jacobina) na Bahia, as macrorregiões norte e noroeste em Minas Gerais, a macrorregião nordeste em Goiás e a cerrados no Piauí fizeram parte desse aglomerado (Figuras 2B e 3B).

A concessão de benefícios mostrou padrão similar quando considerado o sexo dos beneficiários. Na autocorrelação espacial, notou-se aglomerado de padrão alto-alto muito semelhante para os dois sexos, abrangendo 13 macrorregiões de saúde de Minas Gerais, de Goiás, da Bahia e de Tocantins, além do Distrito Federal quando considerado o sexo feminino. Englobaram-se outras 13 macrorregiões de saúde de Minas Gerais, de Goiás, da Bahia, do Piauí e do Distrito Federal, quando considerado o sexo masculino, e 11 delas eram as mesmas em ambos os mapas (Figuras 2C e 3C).

A taxa de concessão de benefícios para indivíduos com idade ≤29 anos foi a mais baixa entre todas as variáveis analisadas. As maiores concentrações de benefícios para indivíduos nessa faixa etária foram observadas em Minas Gerais e na Bahia (Figura 2D). Um aglomerado do tipo alto-alto foi formado englobando a macrorregião norte em Minas Gerais, a nordeste em Goiás e as macrorregiões sudoeste (núcleo básico de saúde – Vitória da Conquista), oeste (núcleo básico de saúde – Barreiras), centro-leste (núcleo regional de saúde – Feira de Santana), centro-norte (núcleo regional de saúde – Jacobina) e norte (núcleo regional de saúde – Juazeiro) na Bahia (Figura 3D). A distribuição de benefícios para indivíduos com idade ≥60 anos mostrou-se mais concentrada no centro do país, com frequências mais elevadas em Goiás e Minas Gerais (Figura 2D). Um extenso aglomerado formou-se entre 15 macrorregiões de saúde de Tocantins, da Bahia, de Minas Gerais, de São Paulo, de Mato Grosso, de Goiás e do Distrito Federal (Figura 3D).

**Figura 2:** Taxa de concessão de benefícios (por 100 mil habitantes) para indivíduos acometidos pela doença de Chagas digestiva segundo tipo de benefício (A), área de residência (B), sexo (C) e idade (D). Brasil, 2004-2016 (n=4.661)

**Figura 3:** Autocorrelação espacial do índice de Moran local para a concessão de benefícios a indivíduos acometidos pela doença de Chagas digestiva segundo tipo de benefício (A), área de residência (B), sexo (C) e idade (D). Brasil, 2004-2016 (n=4.661)

A média de idade no início da doença, assim como a média de idade no início da incapacidade laboral e o tempo médio até a incapacidade laboral, mostrou um padrão de formação de aglomerado mais disperso, esperado devido aos baixos valores apresentados no I de Moran global (Figuras 4A a 4F, Tabela 1). Os aglomerados de padrão baixo-baixo para o tempo médio até a incapacidade laboral foram observados principalmente na região Norte do país. O padrão alto-alto foi observado em 11 macrorregiões de seis estados diferentes – São Paulo, Mato Grosso, Tocantins, Goiás, Pará e Paraná (Figura 3F).

**Figura 4:** Média de idade no início da doença (A), no início da incapacidade (C) e o tempo médio até a incapacidade (E) de beneficiários da seguridade social brasileira acometidos pela doença de Chagas digestiva e autocorrelação espacial do índice de Moran local para a média de idade no início da doença (B), no início da incapacidade (D) e do tempo médio até a incapacidade (F). Brasil, 2004-2016 (n=4.661)

## Discussão

A análise mostrou que, no período 2004-2016, os beneficiários da seguridade social brasileira portadores de doença de Chagas digestiva eram principalmente do sexo masculino, residindo em áreas urbanas, nas regiões Sudeste e Nordeste do país e com idade entre 50 e 59 anos. Macrorregiões de saúde de Minas Gerais, de Goiás e da Bahia se destacaram formando aglomerados do tipo alto-alto na análise do I de Moran local quando a concessão de benefícios foi analisada segundo sexo, área de residência, tipo de benefício concedido e idade. O Distrito Federal também se destacou na composição desses aglomerados, estando ausente apenas nos aglomerados alto-alto relacionados aos moradores de zona rural e com idade ≤29 anos. Os aglomerados do tipo baixo-baixo se formaram principalmente nas regiões Norte e Sul do país.

O uso do banco de dados do Regime Geral da Previdência Social impôs importante limitação ao estudo. A casuística analisada incluiu apenas indivíduos com doença de Chagas inscritos no sistema previdenciário desse regime ou aqueles que possuíam os requisitos básicos para terem direito aos benefícios assistenciais. Essa condição levou à subestimação do número de trabalhadores afetados pela doença, por não estarem incluídos aqueles indivíduos na informalidade, nem os que estavam submetidos a regimes próprios de previdência social. Outra limitação importante se referiu à oportunidade de dados, visto que este estudo possui informações sobre a doença de Chagas na seguridade social apenas até 2016. Embora os dados deste estudo tenham sido coletados há quase uma década, eles são de abrangência nacional e cobrem o período de 12 anos de registros sobre a doença de Chagas digestiva e a seguridade social, servindo de base para análises comparativas sobre o tema em estudos futuros.

A maioria dos estudos relacionados à doença de Chagas tem como foco principal a forma cardíaca da doença. Existe um grande silêncio epidemiológico relacionado à doença de Chagas digestiva e sua real prevalência, a qualidade de vida das pessoas, o seu potencial incapacitante para o trabalho, além de outras questões. Apesar da menor mortalidade da forma digestiva comparada à cardíaca, a morbidade causada pelas manifestações gastrointestinais da doença de Chagas é considerável (10). Houve aumento da mortalidade por doença de Chagas digestiva entre 2000 e 2010. Esse fato pôde estar relacionado à adição de código específico para a doença na décima edição da Classificação Internacional de Doenças (21).

O número de 4.661 benefícios de seguridade social relacionados à doença de Chagas digestiva no período de 12 anos no Brasil é relativamente pequeno. Alguns argumentos podem explicar isso.

1) A prevalência da doença de Chagas digestiva entre os casos de doença de Chagas é 12% (10). Essa prevalência pode estar subestimada, visto que tanto em casos de megaesôfago quanto em casos de megacólon, muitas vezes, o diagnóstico não é realizado (8,9). As pessoas buscam atendimento para o megacólon mais tardiamente do que para o megaesôfago em razão da maior tolerância à obstipação intestinal em relação à disfagia. Muitas pessoas só procuram tratamento quando surgem complicações como fecaloma e volvo (22). Embora o megacólon possa ser mais frequente que o megaesôfago, ele pode não levar a tantas hospitalizações ou pode simplesmente ser mais difícil de diagnosticar. A queixa mais comum do megaesôfago é a disfagia, que pode ocorrer mais cedo no curso da doença ou ser mais perceptível do que a queixa de constipação por megacólon, que, como resultado, acaba sendo menos investigada (4). O estudo da forma digestiva da doença de Chagas em populações rurais, por exemplo, sempre foi dificultado, por conta da falta de aparelhos de raios-X (8).
2) A incidência da doença de Chagas, de forma geral, diminuiu drasticamente nas últimas décadas, não afetando fortemente a população em idade produtiva como em 1960, 1970, 1980 e 1990.
3) A doença de Chagas digestiva é uma manifestação negligenciada de uma doença negligenciada, sem o olhar mais cuidadoso da academia e da saúde pública, o que pode mascarar muitos casos crônicos (4).

Por conta do diagnóstico e do tratamento inadequado e tardio, muitas vezes os indivíduos desenvolvem formas mais graves da doença, acarretando incapacidade laboral. As hospitalizações comumente têm como desfecho as cirurgias de emergência (4). Apesar de os portadores de doença de Chagas digestiva terem sobrevida maior quando comparado com aqueles acometidos pela forma cardíaca, casos mais avançados dos “megas” apresentam alto grau de morbidade e perda considerável da qualidade de vida (23). A doença de Chagas digestiva afeta em geral indivíduos de populações mais vulneráveis, que frequentam os serviços de saúde pública e, com isso, enfrentam grandes desafios no acesso aos cuidados de saúde (24). 

A análise espacial mostrou que os portadores de doença de Chagas digestiva ainda se concentram nas áreas clássicas de transmissão da doença de Chagas – Goiás, Minas Gerais e Bahia. Houve intensa migração de indivíduos infectados do campo para as cidades. Pode-se observar que muitos desses indivíduos se dispersaram pouco, se concentrando ainda nos mesmos territórios, porém em área urbana (11,25,26).

A maioria dos benefícios foi concedida para indivíduos residentes em áreas urbanas. No Brasil, estimou-se em 2014 que 70% a 90% das pessoas infectadas pelo T. cruzi viviam em áreas urbanas, principalmente idosos que nasceram em áreas rurais, onde provavelmente foram infectados (27).

A idade média dos beneficiários foi 49,5±9,3 anos. Esse dado condiz com a clínica da doença, já que, na forma crônica da doença de Chagas digestiva, os indivíduos desenvolvem sintomas entre a terceira e a quinta décadas de vida, com piora ao longo do tempo (4). O controle vetorial bem-sucedido nas décadas de 1980 e 1990 no Brasil reduziu drasticamente a incidência da doença, refletido neste estudo pelo baixo número de beneficiários jovens (11). A Bahia concentrou o maior número de beneficiários abaixo dos 30 anos de idade. Este fato também foi observado entre indivíduos internados por causa do megaesôfago chagásico, onde 82,1% dos casos de indivíduos jovens (menos de 31 anos de idade) eram oriundos da Bahia (28). Esse fato pode estar relacionado às medidas sanitárias de controle dos triatomíneos no Brasil, implementadas em 1975, porém sem cobertura total da área endêmica. Algumas áreas da Bahia, por exemplo, não foram cobertas pelo programa durante os estágios iniciais, e o risco de transmissão nelas permaneceu alto. Somente após 1983, toda a área endêmica para doença de Chagas foi coberta pelo programa nacional de controle vetorial (28,29). 

A concentração da concessão de benefícios em Minas Gerais, em Goiás e na Bahia é resultado da intensa transmissão da doença de Chagas nessas áreas em décadas passadas. A incapacidade laboral da população cronicamente afetada pela doença de Chagas pode ser reduzida se houver acompanhamento longitudinal dos indivíduos, preferencialmente por equipes da Atenção Primária em Saúde, diminuindo o impacto da doença na assistência e na previdência social. 

## Data Availability

O banco de dados e os códigos de análise utilizados na pesquisa estão disponíveis em https://doi.org/10.48331/scielodata.KEK6F (30).
